# Efficacy of Underwater Endoscopic Mucosal Resection for Nonpedunculated Colorectal Polyps: A Systematic Review and Meta-Analysis

**DOI:** 10.7759/cureus.17261

**Published:** 2021-08-17

**Authors:** Takeshi Yamashina, Noboru Hanaoka, Takeshi Setoyama, Jun Watanabe, Masahiro Banno, Hiroyuki Marusawa

**Affiliations:** 1 Gastroenterology and Hepatology, Osaka Red Cross Hospital, Osaka, JPN; 2 Gastroenterology and Hepatology, Kansai Medical University Medical Center, Moriguchi, JPN; 3 Division of Gastroenterological, General and Transplant Surgery, Jichi Medical University, Shimotsuke, JPN; 4 Department of Systematic Reviewers, Systematic Review Workshop Peer Support Group (SRWS-PSG), Osaka, JPN; 5 Department of Psychiatry, Nagoya University Graduate School of Medicine, Nagoya, JPN; 6 Department of Psychiatry, Seichiryo Hospital, Nagoya, JPN

**Keywords:** underwater emr, conventional endoscopic mucosal resection (cemr), colon cancer prevention, colorectal polyp, systematic review and meta-analysis

## Abstract

Recently, underwater endoscopic mucosal resection (UEMR) without submucosal injection was introduced as a new replacement for conventional EMR (CEMR) and was reported to be useful for resecting large colonic polyps. Here, we aimed to assess the efficacy and safety of these two methods by a systematic review and meta-analysis.

We comprehensively searched multiple databases until July 2021 to identify randomized controlled trials (RCTs) comparing UEMR with CEMR. The primary outcomes were the proportion of R0 resection and mean procedure time, and the secondary outcomes were the proportion of en bloc resection and all adverse events. Three reviewers independently searched for articles, extracted data, and assessed the risk of bias. We evaluated the certainty of evidence using the Grading of Recommendations, Assessment, Development, and Evaluation approach. This study was registered in www.protocols.io (Protocol Integer ID: 40849).

We included six RCTs (1,374 polyps). We judged that a meta-analysis was not available, and the data were summarized narratively for the proportion of R0 resection. Regarding procedure time, UEMR likely resulted in a large reduction (mean difference = −64.3 seconds; 95% confidence interval (CI) = −122.5 to −6.0 seconds; I^2 ^= 86%; moderate certainty of evidence). UEMR likely resulted in a large increase in en bloc resection (odds ratio = 1.85; 95% CI = 1.15 to 2.98; I^2 ^= 60%; moderate certainty of evidence). Percentages of adverse events were 0-17% with CEMR and 0-16% with UEMR.

In summary, UEMR might have higher efficacy than CEMR in the endoscopic resection of nonpedunculated colorectal polyps, with likely a large reduction in procedure time.

## Introduction and background

Colorectal cancer is the third most common cancer (the third most commonly occurring cancer in men and the second most commonly occurring cancer in women) worldwide, and the second cancer with higher mortality. More than 1.9 million new colorectal cancer cases and 935,000 deaths were estimated to occur in 2020 [1]. Despite the multiple factors involved in colorectal cancer development, a good prognosis can be expected if the disease is detected and treated at an early stage. Endoscopic mucosal resection (EMR), widely used in the prevention of colorectal cancer, is performed by injecting fluid under the colonic mucosa at the site of the lesion to prevent excessive coagulation of the colonic wall caused by resection using electrocautery [2]. However, some proficiency in injecting and snaring is required. In addition, as polyp size increases, en bloc resection becomes difficult, which results in a piecemeal resection that can lead to local recurrence, introducing a further clinical problem [3].

Endoscopic submucosal dissection (ESD) facilitates the excision of large superficial colorectal lesions in an en bloc fashion. Furthermore, ESD can achieve a higher en bloc resection rate than EMR, regardless of tumor size or location [4]. However, technical difficulties and being more time-consuming and costly than EMR prevent the adoption of colorectal ESD as one of the standard endoscopic therapies.

Recently, underwater EMR (UEMR) without submucosal injection, as described by Binmoeller et al., was introduced as a new alternative to conventional EMR (CEMR) and has been reported useful for removing large colonic polyps [5]. In UEMR, the bowel lumen is flooded with water instead of air/CO_2_, and the polyp is removed with no injection. Compared with CEMR, UEMR makes it easier to snare large colorectal polyps, residual recurrent lesions, and rectal neuroendocrine tumors and has the potential to improve treatment outcomes [6,7]. Randomized controlled trials (RCTs) have reported a significantly higher proportion of R0 resection for UEMR [8] and a significantly shorter procedure time than with CEMR [9].

Although several systematic reviews and meta-analyses of UEMR have already shown efficacy and safety, the superiority of UEMR over CEMR has not been sufficiently examined because it has not been compared with CEMR [10] or has been compared with CEMR in a mixture of cohort studies and RCTs [11-14]. RCTs are the most effective way to scientifically validate new medical interventions. Aside from one study by Tziatzios et al., we are unaware of any other meta-analyses of RCTs [15]. Therefore, we excluded cohort studies and conducted a meta-analysis involving additional RCTs.

## Review

Reporting guidelines

This systematic review was conducted in accordance with the Preferred Reporting Items for Systematic Reviews and Meta-Analyses (PRISMA) guidelines [16]. The study group also followed the recommendations listed in the Cochrane Handbook (Appendix 1) [17]. We published our review protocol in www.protocols.io [18].

Criteria for considering studies for this review

Types of Studies

We included RCTs that assessed the efficacy of UEMR compared with CEMR for endoscopic removal of colorectal polyps. We did not apply language or country restrictions and included all published and unpublished articles, abstracts of conferences, and letters. We excluded duplicate publications, quasi-RCTs, cluster-randomized trials, crossover studies, quasi-experimental designs, case reports, and nonhuman studies. We did not exclude studies based on the observation length or year of publication. This study involved men and women aged 18 years or older who underwent UEMR or CEMR for colorectal polyps, and excluded participants with UEMR without submucosal injection, pedunculated lesions based on the Paris endoscopic classification [19]; residual or recurrent lesions after endoscopic resection; and colitis ulcer, Crohn’s disease lesions, and familial adenomatous polyposis.

Types of Intervention

The intervention was UEMR with complete deflation of the colorectal lumen air/CO_2_ and total immersion of the polyp in water. Following this, the polyp and the surrounding mucosa were snared and removed with electrocautery. The comparator was CEMR with needle injection of water into the submucosa, followed by entrapment of the mucosal protrusion with a snare and resection using electrocautery.

Types of outcome measures

Primary Outcomes

The primary outcomes are the proportion of R0 resection and mean procedure time. The proportion of R0 resection was calculated as the number of R0 resections divided by the total number of polyps. R0 resection was defined as en bloc resection with a confirmed negative resection margin on histology. En bloc resection was defined as the removal of one polyp section that was assessed endoscopically. The procedure time for the UEMR group was calculated from the beginning of immersion in water through the endoscope until the polyp was completely removed. The procedure time for the CEMR group was calculated from the insertion of the injection needle or injection until the polyp was completely removed.

Secondary Outcomes

The secondary outcomes were the proportion of en bloc resection and adverse events. The proportion of en bloc resection was calculated as the number of en bloc resections divided by the total number of polyps. Definitions of adverse events were set by the original authors.

Search methods to identify studies

Electronic Searches

We searched the electronic databases, Cochrane Central Register of Controlled Trials (CENTRAL), Medical Literature, Analysis and Retrieval System Online (MEDLINE, Ovid, 1946 to August 2020), Excerpta Medica database (EMBASE, ProQuest, 1974 to August 2020), search portal of the World Health Organization International Clinical Trials Registry Platform (ICTRP), and ClinicalTrials.gov (Appendix 2-6 available at https://data.mendeley.com/datasets/ph9hwfn5v4/1) for ongoing or unpublished trials. We updated the electronic searches on July 15, 2021. The keywords used in our search were a combination of “polyps” OR “neoplasms” OR “intestines” OR “colorect*” OR “colon*” OR “bowel” OR “rect*” OR “intestinal mucosa” AND “polyp*” OR “neoplas*” OR “tumour*” OR “tumor” OR “adenom*” OR “lesion*” OR “carcinom*” OR “adenocarcinom*” OR “cancer*” AND “endoscopic mucosal resection” OR “polypectomy” OR “EMR” OR “endoscopic resection” OR “endoscopic mucosectom*” AND “underwater” OR “under water” AND “randomized controlled trial” OR “controlled clinical trial” OR “randomized” OR “placebo” OR “drug therapy” OR “randomly” OR “trial” OR “groups.” We checked the reference lists of the identified studies, including international guidelines [20-23], the reference lists of eligible studies, and articles citing eligible studies. We consulted the authors of original studies for data not yet published and for supplementary data. If published studies were duplicated, only the latest version, or at least the more complete version, was reviewed.

Data collection and analysis

Study Selection

Three reviewers (TY, TS, and NH) independently checked titles and abstracts and then assessed eligibility based on the full texts identified in the search. If relevant data could not be found, we contacted the original authors. Disagreements between the reviewers were settled by discussion, and if consensus could not be reached, two additional reviewers (JW and MB) acted as arbiters.

Data Extraction and Management

Three reviewers independently extracted data from the included studies using a standardized data collection form that was pre-evaluated using 10 randomly selected studies. The form comprised information regarding the study design, study population, patient characteristics (sex, mean age, number of patients undergoing UEMR and CEMR, number of polyps, mean polyp size, polyp location, and histological type of polyp), proportion of R0 resection with UEMR and CEMR, proportion of en bloc resection with UEMR and CEMR, mean procedure time for UEMR and CEMR, and percentage of adverse events (bleeding and perforation). Any disagreements were resolved by discussion, and if this failed, two additional reviewers (JW and MB) acted as arbiters.

Assessment of Risk of Bias in the Included Studies

Three reviewers (TY, TS, and NH) independently assessed the risk of bias with the Risk of Bias 2 tool [24]. Disagreements between the three reviewers were discussed, and if consensus could not be achieved, two additional reviewers (JW and MB) acted as arbiters. If a meta-analysis was not feasible, the data were summarized narratively to rate the certainty in evidence using the Grading of Recommendations, Assessment, Development, and Evaluations (GRADE) approach according to a previous report and the synthesis without meta-analysis (SWiM) guideline [25,26].

Measures of Treatment Effects

We pooled the relative risk ratios and 95% confidence intervals (CIs) for the variables, R0 resection, and en bloc resection. We pooled the mean differences (MDs) and the 95% CIs for the continuous variables, UEMR procedure time, and CEMR procedure time. If the included studies used several different scales, we pooled the effect estimates with standard MDs.

Unit of Analysis Issues

For continuous data, only the sample size was reduced; means and standard deviations remained unchanged [24]. For multiple comparisons, we included all intervention groups relevant to this review.

Missing Outcomes

Intention-to-treat analyses were performed for all dichotomous data, as much as possible. We did not impute missing data for continuous data, in accordance with the recommendation in the Cochrane Handbook [17]. We performed a meta-analysis using the data from the original studies. We asked the original authors for data not presented.

Assessment of Heterogeneity

Statistical heterogeneity was assessed by visually inspecting the forest plots and calculating the I^2^ statistic (I^2^ values: 0-40%, might not be important; 30-60%, may represent moderate heterogeneity; 50-90%, may represent substantial heterogeneity; 75-100%, considerable heterogeneity) [17]. When there was substantial heterogeneity (I^2^ > 50%), we assessed the reason for the heterogeneity. The Cochrane Chi^2^ test (Q-test) was performed for the I^2^ statistic, and p values of less than 0.10 were defined as statistically significant.

Assessment of Reporting Bias

We searched the clinical trials registry system (ClinicalTrials.gov and ICTRP) and performed an extensive literature search for unpublished trials. Although we planned to evaluate potential publication bias by visual inspection of the funnel plot, we did not conduct this test because we found fewer than 10 trials.

Meta-Analysis

We performed the meta-analysis using Review Manager software (RevMan 5.4) and a random-effects model. Some guidelines recommend ESD rather than EMR for nonpedunculated colorectal polyps larger than 20 mm [20,21]. Therefore, we evaluated the ad hoc subgroup analyses of the lesion size (≤20 mm or >20 mm) to determine the influence of effect modifiers on the results. And as a difference between the protocol and the review, we were unable to perform other prespecified subgroup analyses and sensitivity analyses for R0 resection and procedure time.

Search results

**Figure 1:**
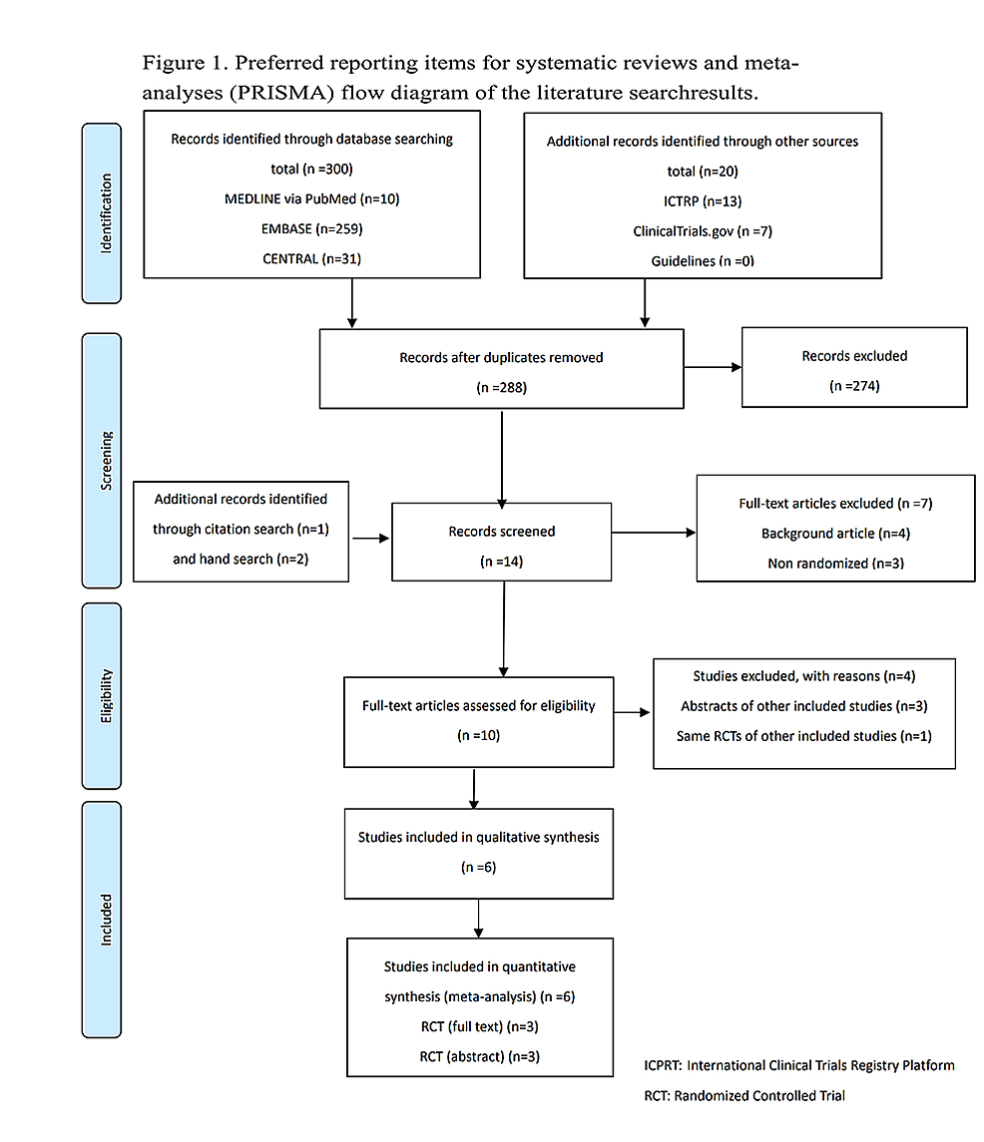
PRISMA flow diagram of the literature search results. PRISMA: Preferred Reporting Items for Systematic Reviews and Meta-Analyses

**Figure 2:**
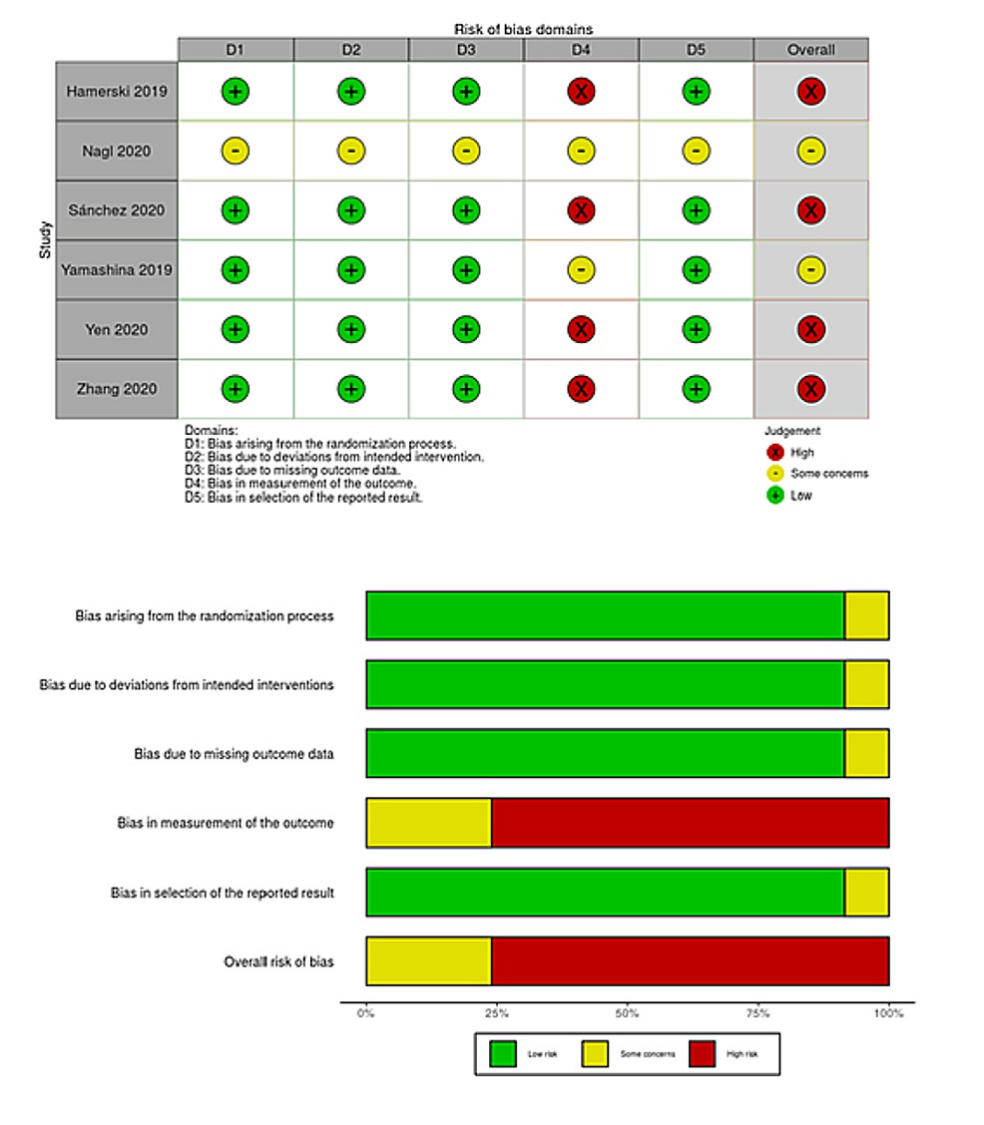
Risk-of-bias table and risk-of-bias graph for R0 resection.

**Figure 3:**
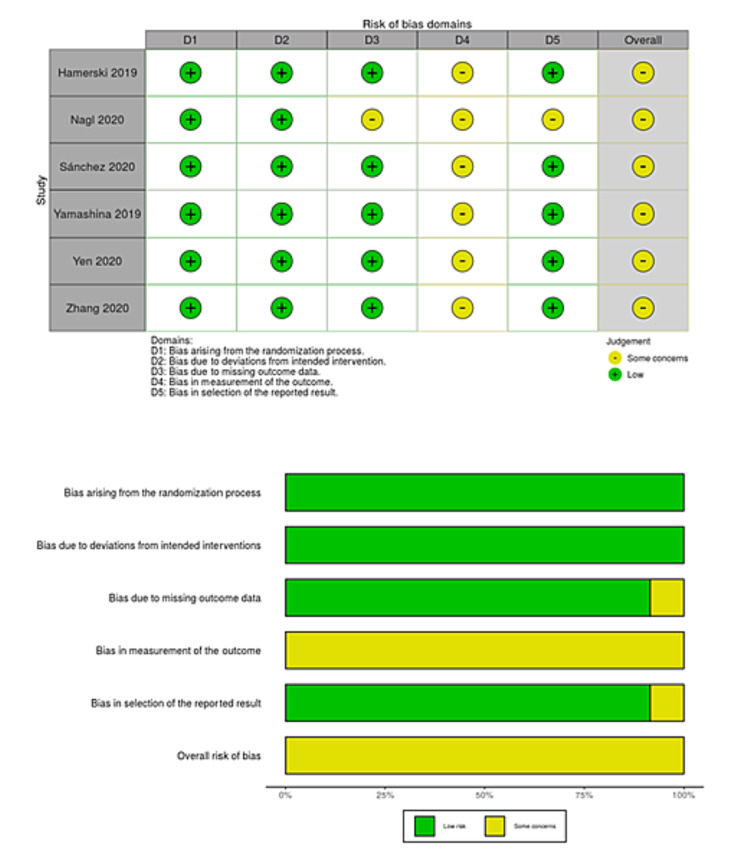
Risk-of-bias table and risk-of-bias graph for en bloc resection.

**Figure 4:**
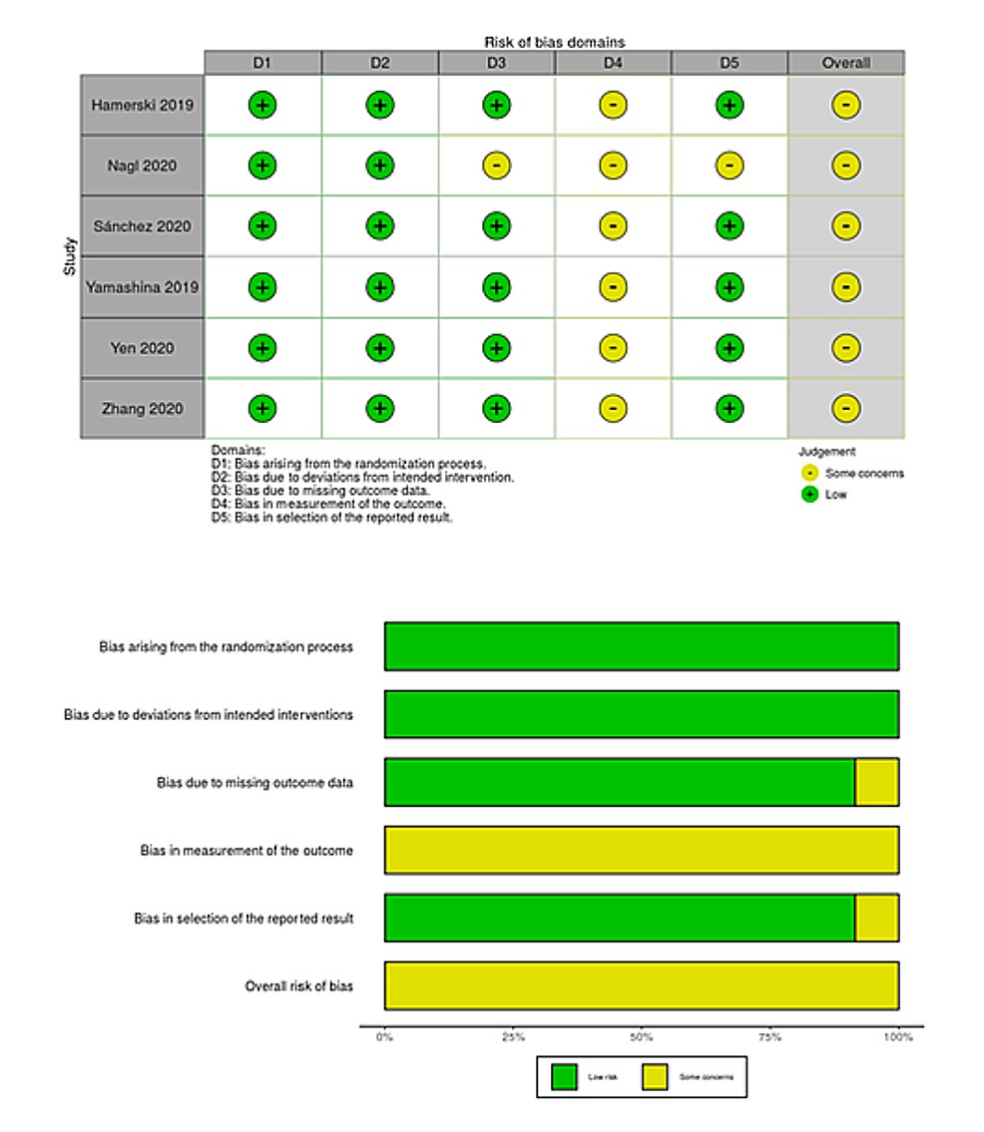
Risk-of-bias table and risk-of-bias graph for procedure time.

**Figure 5:**
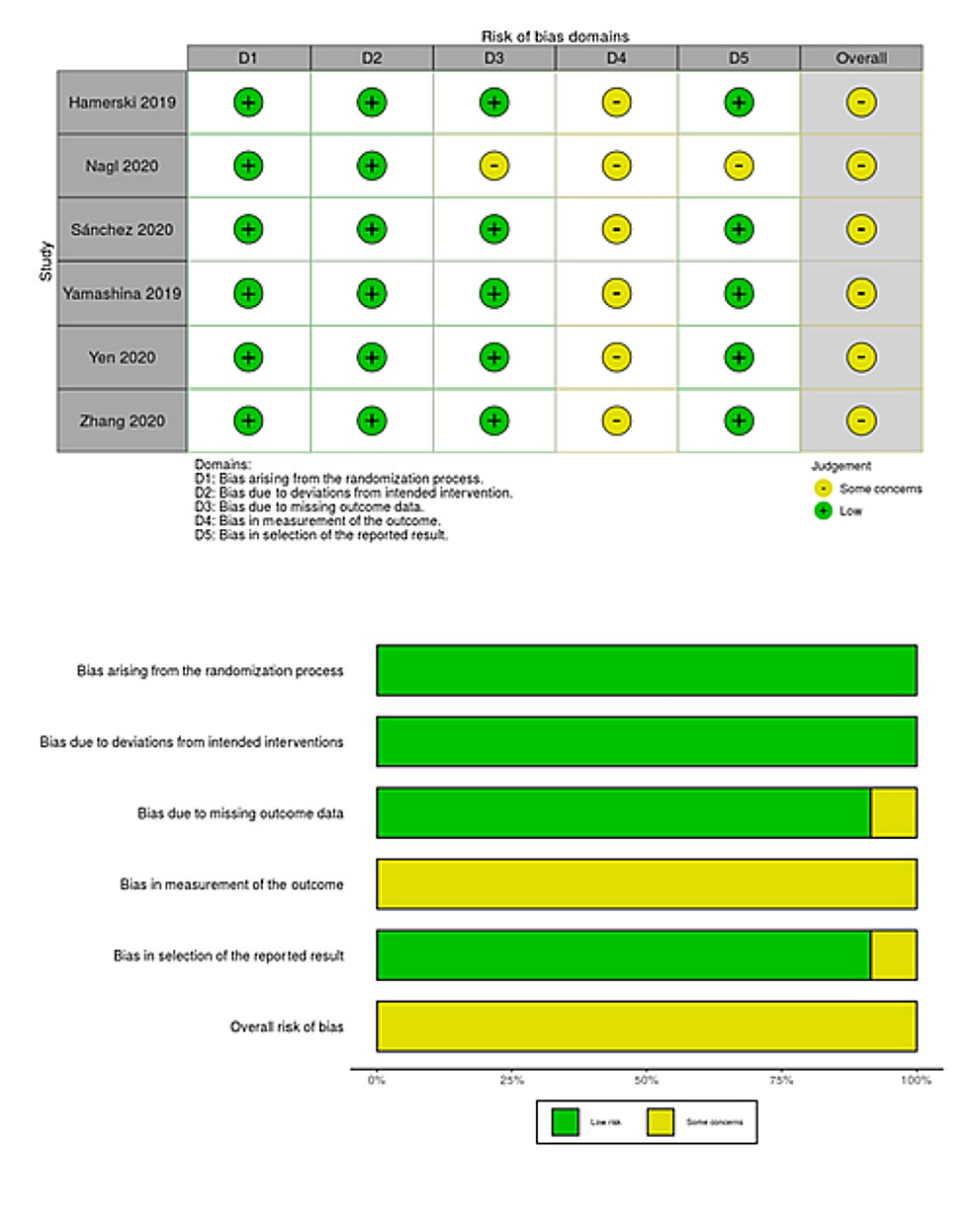
Risk-of-bias table and risk-of-bias graph for adverse events.

We searched a total of 218 studies on August 15, 2020 and updated the electronic searches on July 15, 2021 (Figure 1). We finally included six studies with 1,374 polyps that met our inclusion criteria, 675 of which were removed by CEMR and 699 by UEMR [8,9,27-30]. Two studies included three pedunculated polyps [8,30], so these polyps were excluded from the meta-analysis. However, the summary of the characteristics included these data. Three studies were original articles [8,9,30], and the remaining three were abstracts only [27-29]. Table 1 shows a summary of the characteristics of the included studies. The risk of bias for each study is presented in Figures 2-5. Overall, four studies had a high risk of bias as these showed bias in the outcomes measurement. We included all six studies summarized narratively with the primary outcome of the proportion of R0 resection, and meta-analyses with the primary outcome of procedure time and secondary outcomes [8,9,27-30]. We found no unpublished studies.

**Table 1:** Summary of the characteristics of the included studies. NS: not stated; UEMR: underwater endoscopic mucosal resection; CEMR: conventional endoscopic mucosal resection; IQR: interquartile range *: inclusion criteria, †: abstract

Study	Setting	Enrollment, n (patients/polyps) (male/female)	Number of polyps (CEMR/UEMR)	Polyp size (CEMR/UEMR)	Polyp morphology (CEMR/UEMR) (%)
Hamerski et al. (2018) [27]	USA, Multi-center	178/179 NS	88/91	(mm, mean, range) 28.1 (15-70)/29 (15-50)	IIa/IIb 69 IIc+IIa/IIb or IIc 2 Is+ IIa/IIb or Is 30
Nagl et al. (2020) [29]	Germany, Single-center	NS/117 NS	59/58	(mm, range) 20-40*	Large sessile or flat colonic polyps*
Sánchez et al. (2020) [28]	Spain, Multi-center	NS/267 NS	141/126	32.8^†^	NS
Yamashina et al. (2019) [8]	Japan, Multi-center	214/214 139/71	102/108	(mm, median, range) 13.5 (7–25)/14 (7-25)	IIa 57/59 IIc 0/0.9 Is 43/38 Ip 0/1.9
Yen et al. (2020) [9]	USA, Single-center	255/462 248/7	214/248	(mm, mean, range) 9.9 (6-45)/9.9 (6-40)	Is 50.8/53.7 IIa 43.2/41.1 IIb 4.4/1.9 IIc 0/0.5 Mixed 0/0.5
Zhang et al. (2020) [30]	China, Multi-center	130/142 75/55	71/71	(mm, median, IQR) 5.0 (4.0–7.0)/6.0 (5.0–8.0)	Is 70.4/76.1 Ip 0/1.4 IIa 29.6/22.5

Primary outcomes

R0 Resection

Four studies reported complete resections [9,27,28,30] and one reported R0 resections [8] from a total of 1,257 polyps. Although complete resection was often used as a synonym for R0 resection, each study had different definitions: one study defined R0 resection as en bloc resection with histologically confirmed negative lateral and vertical resection margins [8], one defined R0 resection as complete en bloc resection of a polyp with tumor-free lateral margins confirmed by negative biopsy [30], one defined it as pathological assessment of biopsy specimens from the resection margin [9], and another did not provide definitions. Therefore, we judged that a meta-analysis was not feasible, and the data were summarized narratively to rate the certainty in evidence using the GRADE approach (Tables 2, 3). Evidence derived from three RCTs showed that the proportion of R0 resection in the UEMR group was higher than that in the CEMR group [8,27,28], while two reported no difference [9,30]. Our considered judgment was low certainty (rated down for very serious limitations in study design).

**Table 2:** Summary of findings: UEMR compared to CEMR for patients with colorectal polyp. *The risk in the intervention group (and its 95% CI) is based on the assumed risk in the comparison group and the relative effect of the intervention (and its 95% CI). ^a^Downgraded one level for very serious limitations in study design (allocation concealment which did not mask outcome assessment in all studies); ^b^Downgraded one level for very serious limitations in study design (surrogate outcomes are used in four studies; ^c^Downgraded one level for very serious limitations in small sample size GRADE Working Group grades of evidence. High certainty: We are very confident that the true effect lies close to that of the estimate of the effect. Moderate certainty: We are moderately confident in the effect estimate. The true effect is likely to be close to the estimate of the effect, but there is a possibility that it is substantially different. Low certainty: Our confidence in the effect estimate is limited. The true effect may be substantially different from the estimate of the effect. Very low certainty: We have very little confidence in the effect estimate. The true effect is likely to be substantially different from the estimate of effect. CI: Confidence interval; OR: Odds ratio; MD: Mean Difference; UEMR: Underwater endoscopic mucosal resection; CEMR: Conventional endoscopic mucosal resection; GRADE: Grading of Recommendations Assessment, Development, and Evaluation

Outcomes	Anticipated absolute effects^*^ (95% CI)		Relative effect (95% CI)	Number of polyps (studies)	Certainty of the evidence (GRADE)	Comments
	Risk with CEMR	Risk with UEMR				
R0 resection: Assessed using a variety of scales	Three studies showed that the R0 resection rate in the UEMR group was higher than that in the CEMR group			1,257 (5 RCTs)	⨁⨁◯◯ LOW ^a,b^	UEMR may result in a slight increase in R0 resection
En bloc resection	582 per 1,000	721 per 1,000 (616 to 806)	OR: 1.84 (1.14 to 2.96)	1,374 (6 RCTs)	⨁⨁⨁◯ MODERATE ^a^	UEMR likely results in a large increase in en bloc resection
Procedure time	The mean procedure time was 81 to 1,793 seconds	MD 64.5 seconds lower (-122.9 lower to -6.1 lower)	-	1,257 (5 RCTs)	⨁⨁⨁◯ MODERATE ^a^	UEMR likely results in a decrease in procedure time
Adverse event	There were 0-17% adverse events with the CEMR and 0-16% adverse events with the UEMR			1,374 (6 RCTs)	⨁⨁◯◯ LOW ^a,c^	UEMR may result in a slight decrease in an adverse event

**Table 3:** Rating the certainty in the evidence of R0 resection. GRADE: Grading of Recommendations Assessment, Development, and Evaluation; RRR: relative risk reduction

GRADE domain	Judgment	Concerns about certainty domains
Risk of bias	Downgraded one level for serious limitations in study design (allocation concealment which did not mask outcome assessment in all studies)	Serious
Inconsistency	The patients, intervention, and comparators in the studies provide direct evidence to the clinical question at hand	Not serious
Indirectness	Downgraded one level for serious limitations in study design (surrogate outcomes are used in four studies)	Serious
Imprecision	The total number of patients included in all the trials was 1,260 and the total number of events was 190. Although the number of events was relatively small, we assumed RRR as 50%. Therefore, we judged the evidence was not imprecise	Not serious
Publication bias	We did not strongly suspect publication bias because both negative and positive trials were published, and the search for studies was comprehensive	Undetected

Procedure Time

**Figure 6:**
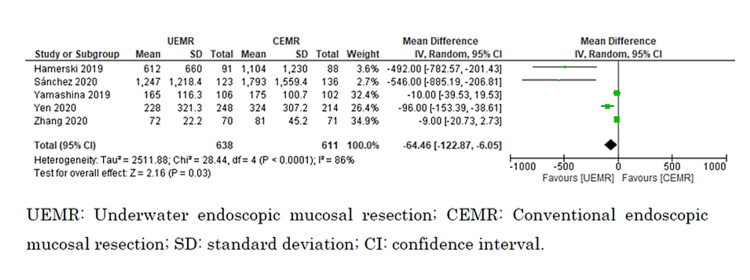
Forest plot comparing procedure time between UEMR and CEMR.

Five studies reported procedure time [8,9,27,28,30]. We could include a total of 1,257 polyps in our analysis. UEMR likely resulted in a large reduction in procedure time, although there was significant heterogeneity (MD = −64.5 seconds; 95% CI = −122.9 to −6.1 seconds; I^2^ = 86%; moderate certainty of the evidence) (Figure 6).

Secondary outcomes

En Bloc Resection

**Figure 7:**
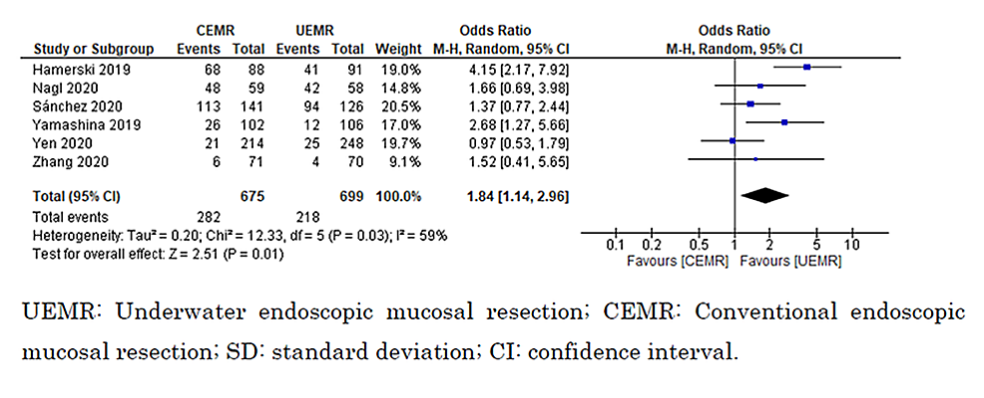
Forest plot comparing en bloc resection rate between UEMR and CEMR.

All six studies reported en bloc resection [8,9,27-30]. We could include a total of 1,374 polyps in our analysis. UEMR likely resulted in a large increase in the proportion of en bloc resection (odds ratio = 1.84; 95% CI = 1.14 to 2.96; I^2^ = 59%; moderate certainty of the evidence) (Figure 7).

All Adverse Events

All six studies reported all adverse events and included a total of 1,374 polyps [8,9,27-30]. One study reported no adverse events [9], and five studies reported adverse events, and almost all of them were delayed bleeding [8,27-30] (Table 2). There were 0-17% adverse events with CEMR and 0-16% adverse events with UEMR.

Subgroup Analysis

**Figure 8:**
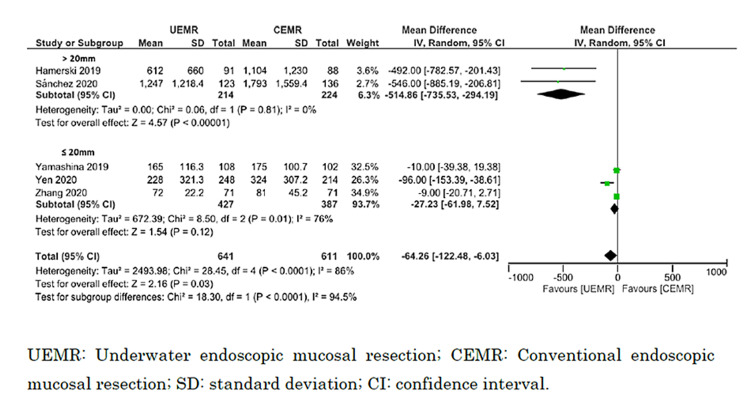
Forest plot comparing procedure time between UEMR and CEMR on the size of the lesion (≤20 or >20 mm).

**Figure 9:**
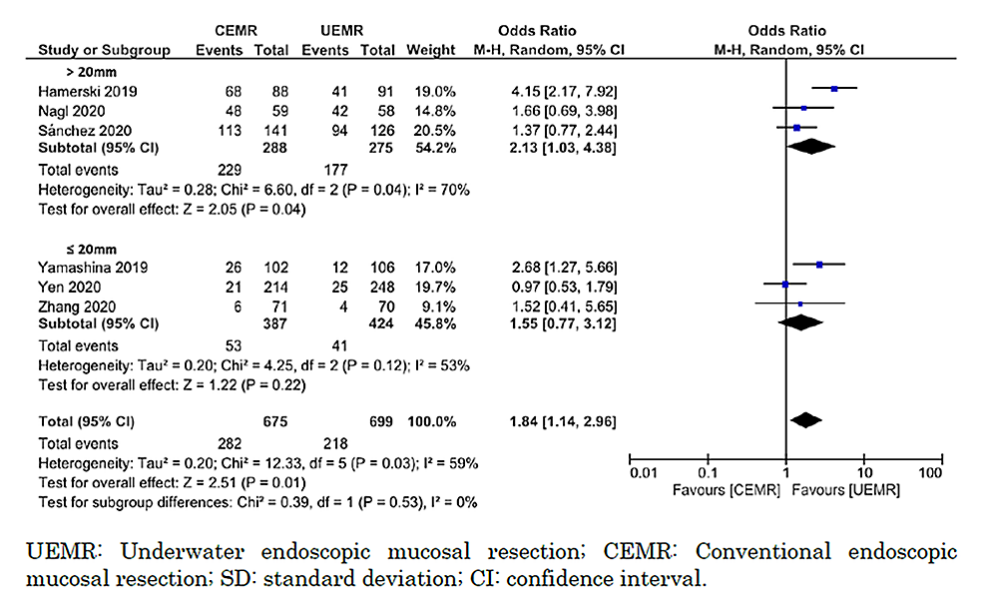
Forest plot comparing en bloc resection between UEMR and CEMR on the size of the lesion (≤20 or >20 mm).

Subgroup analyses indicated that procedure time was significantly shorter for lesions measuring >20 mm versus ≤20 mm (I^2^ = 94.5%; p < 0.0001; chi^2^ = 18.3), and the procedure time for UEMR was significantly shorter than for CEMR for lesions >20 mm (MD = −514.9 seconds; 95% CI = −735.5 to −294.2 seconds; I^2^ = 0%) but not for lesions ≤20 mm (MD = −27.2 seconds; 95% CI = −62.0 to 7.5 seconds; I^2^ = 76%). However, there was no significant difference in the rates of en bloc resection between lesions >20 mm and ≤20 mm (I^2^ = 0%; p = 0.53; chi^2^ = 0.39; I^2^ = 0%). UEMR showed a significant increase in the rate of en bloc resection for lesions >20 mm compared with CEMR (odds ratio = 2.13; 95% CI = 1.03 to 4.38; I^2^ = 70%) but not for lesions ≤20 mm (odds ratio = 1.55; 95% CI = 0.77 to 3.12; I^2^ = 53%) (Figures 8, 9).

Discussion

This review of six RCTs and 1,374 polyps found that UEMR might have better efficacy than CEMR in endoscopic resection for nonpedunculated colorectal polyps. Although several systematic reviews and meta-analyses have already been published and have reported UEMR as effective for the resection of flat colorectal polyps, their data are insufficient because the results were derived from only a single-arm UEMR trial [10] or a mixture of cohort trials and RCTs [11-14].

Tziatzios et al. showed that the rate of en bloc resection was significantly higher in the UEMR group in their meta-analysis; however, there was no significant difference in the complete resection rate between the UEMR and CEMR groups, and these results were divergent [15]. We judged that a meta-analysis was not feasible and summarized the data narratively for our primary outcome, the proportion of R0 resection. The reasons for doing so were that each study had different definitions of R0 resection (we regarded complete resection as a synonym) with the major difference related to whether the negative resection margin was confirmed endoscopically or histologically. Another difference was whether histological confirmation was achieved using a complete en bloc resected specimen, piecemeal resected specimen, or biopsy specimen near the polyp. Of course, histological assessment of en bloc resected specimens is the most desirable method for confirmation. However, as the polyp size increases, it becomes more difficult to obtain complete en bloc resection, and multiple cases are needed to conduct RCTs. In the present study, the UEMR group had higher efficacy than the CEMR group in three RCTs [8,27,28] while another two showed no difference [9,30]. These two studies mainly targeted small polyps less than 10 mm in size. Our subgroup analysis also showed that UEMR was better than CEMR regarding the proportion of en bloc resection rate and procedure time for colorectal polyps >20 mm. The proportion of R0 resection is substantial for small polyps, with little benefit being derived from water immersion. If the polyp size is larger than 10 mm, however, R0 resection becomes less successful [20]. Because these cases may result in local recurrence after piecemeal resection, UEMR is considered clinically important for polyps larger than 10 mm.

In this study, we found that UEMR might be associated with a higher proportion of en bloc resection and shorter procedure time than CEMR. In line with four previous systematic reviews and meta-analyses demonstrating that UEMR was associated with a statistically significantly higher proportion of en bloc resection than CEMR [11-14], our results also showed that UEMR likely results in a large increase in en bloc resection. UEMR was first reported by Binmoeller et al. [5], and water immersion, the main point of UEMR, is thought to have several advantages that support our results, namely, the colorectal wall extension force is decreased, which is accompanied by increased mucosal shrink and flotation upward into the lumen. This results in flat mucosal lesions becoming polypoid, which makes snaring easier and permits eventual total en bloc resection.

Although previous systematic reviews and meta-analyses have not clearly analyzed the comparison of the procedure time for UEMR versus CEMR, our results showed that UEMR likely results in a large reduction in procedure time. Submucosal injection requires complicated maneuvers and several steps before resection, such as good positional control of the colonoscope to the polyp, insertion of the injection needle into the colonoscope, correct injection into the submucosa, creation of sufficient submucosal fluid elevation, and removal of the injection needle, which are time-consuming. In contrast, UEMR requires only deflation of air/CO_2_ and injection of water into the lumen. In addition to the advantages already mentioned, water immersion minimizes luminal distension and flexure angulation, which improves endoscopic maneuverability. For these reasons, UEMR is likely to save time.

As CEMR has already been broadly generalized for resecting colorectal polyps in daily clinical settings, it should also be possible to generalize UEMR. Submucosal injection can create a cushion in the submucosal layer and enhance the safety of hot snare polypectomy by reducing deep thermal injury [2], which is thought to be a potential risk factor for adverse events. Therefore, considering submucosal injection prior to hot snare polypectomy is recommended [20], and CEMR has become very popular because of this safety aspect. However, Binmoeller et al. demonstrated with endosonographic observation that with underwater endoscopy, deflation of air/CO_2_ and immersion in water ensure safety by separating the mucosa and submucosa away from the muscularis propria [5]. Submucosal injection requires a considerable level of proficiency to avoid misguidance into the muscularis propria, poor elevation for snaring, and formation of a hematoma. The underwater procedure, however, is relatively easy to learn because it requires only deflation of air/CO_2_ and injection of water into the lumen [5]. Therefore, UEMR is potentially easily generalizable to the daily clinical setting.

Several limitations of this study must be mentioned. First, four of the six studies, three of which were in abstract form, showed a high risk of bias in the selection of the reported results owing to the lack of a pre-registered protocol or no pre-specified outcomes. Nevertheless, we asked the original authors for protocol and prespecified outcomes as much as possible and evaluated the data to the maximum extent possible. Second, the recurrence rate was inadequately evaluated in the six eligible RCTs. Two of the six studies did not evaluate long-term follow-up and recurrence, and the follow-up rates of the other four studies were not high enough. Therefore, a well-designed follow-up study of local recurrence after UEMR and CEMR is warranted. Third, the participating endoscopists were unblinded to the group allocations. Endoscopists perform UEMR or CEMR according to the allocation results; therefore, this problem is unavoidable in RCTs.

## Conclusions

This systematic review and meta-analysis showed that UEMR might have higher efficacy than CEMR in endoscopic resection of nonpedunculated colorectal polyps, with a probable large reduction in procedure time and increased proportion of en bloc resection. The findings suggest that endoscopists might preferably perform UEMR rather than CEMR for nonpedunculated colorectal polyps. However, meta-analysis for R0 resection was not feasible in this study; therefore, further high-quality RCTs are needed.

## Appendices

**Table 4:** PRISMA 2009 checklist. PRISMA: Preferred Reporting Items for Systematic Reviews and Meta-Analyses

Section/topic	#	Checklist item	Reported on page number	
TITLE				
Title	1	Identify the report as a systematic review, meta-analysis, or both	1	
ABSTRACT				3,4
Structured summary	2	Provide a structured summary including, as applicable: background; objectives; data sources; study eligibility criteria, participants, and interventions; study appraisal and synthesis methods; results; limitations; conclusions and implications of key findings; systematic review registration number	3,4	
INTRODUCTION				
Rationale	3	Describe the rationale for the review in the context of what is already known	5	
Objectives	4	Provide an explicit statement of questions being addressed with reference to participants, interventions, comparisons, outcomes, and study design (PICOS)	5	
METHODS				
Protocol and registration	5	Indicate if a review protocol exists, if and where it can be accessed (e.g., Web address), and, if available, provide registration information including registration number	6	
Eligibility criteria	6	Specify study characteristics (e.g., PICOS, length of follow-up) and report characteristics (e.g., years considered, language, publication status) used as criteria for eligibility, giving rationale	6,7	
Information sources	7	Describe all information sources (e.g., databases with dates of coverage, contact with study authors to identify additional studies) in the search and date last searched	8	
Search	8	Present full electronic search strategy for at least one database, including any limits used, such that it could be repeated	8 Appendix 2	
Study selection	9	State the process for selecting studies (i.e., screening, eligibility, included in the systematic review, and, if applicable, included in the meta-analysis)	7	
Data collection process	10	Describe the method of data extraction from reports (e.g., piloted forms, independently, in duplicate) and any processes for obtaining and confirming data from investigators	7,8	
Data items	11	List and define all variables for which data were sought (e.g., PICOS, funding sources) and any assumptions and simplifications made	9,10	
Risk of bias in individual studies	12	Describe methods used for assessing the risk of bias of individual studies (including specification of whether this was done at the study or outcome level), and how this information is to be used in any data synthesis	10,11	
Summary measures	13	State the principal summary measures (e.g., risk ratio, difference in means)	10	
Synthesis of results	14	Describe the methods of handling data and combining results of studies, if done, including measures of consistency (e.g., I^2^) for each meta-analysis	11	
